# A method for capturing dynamic spectral coupling in resting fMRI reveals domain-specific patterns in schizophrenia

**DOI:** 10.3389/fnins.2023.1078995

**Published:** 2023-04-27

**Authors:** Deniz Alaçam, Robyn Miller, Oktay Agcaoglu, Adrian Preda, Judith Ford, Vince Calhoun

**Affiliations:** ^1^Tri-institutional Center for Translational Research in Neuroimaging and Data Science (TReNDS), Georgia State, Georgia Tech, Emory, Atlanta, GA, United States; ^2^Department of Mathematics, Bursa Uludag University, Bursa, Türkiye; ^3^Department of Psychiatry and Human Behavior, University of California, Irvine, Irvine, CA, United States; ^4^San Francisco VA Medical Center, University of California, San Francisco, San Francisco, CA, United States; ^5^Department of Psychiatry, University of California, San Francisco, San Francisco, CA, United States

**Keywords:** resting state—fMRI, dynamic spectral coupling, schizophrenia, visual network, FBIRN

## Abstract

**Introduction:**

Resting-state functional magnetic resonance imaging (rs-fMRI) is a powerful tool for assessing functional brain connectivity. Recent studies have focused on shorter-term connectivity and dynamics in the resting state. However, most of the prior work evaluates changes in time-series correlations. In this study, we propose a framework that focuses on time-resolved spectral coupling (assessed via the correlation between power spectra of the windowed time courses) among different brain circuits determined via independent component analysis (ICA).

**Methods:**

Motivated by earlier work suggesting significant spectral differences in people with schizophrenia, we developed an approach to evaluate time-resolved spectral coupling (trSC). To do this, we first calculated the correlation between the power spectra of windowed time-courses pairs of brain components. Then, we subgrouped each correlation map into four subgroups based on the connectivity strength utilizing quartiles and clustering techniques. Lastly, we examined clinical group differences by regression analysis for each averaged count and average cluster size matrices in each quartile. We evaluated the method by applying it to resting-state data collected from 151 (114 males, 37 females) people with schizophrenia (SZ) and 163 (117 males, 46 females) healthy controls (HC).

**Results:**

Our proposed approach enables us to observe the change of connectivity strength within each quartile for different subgroups. People with schizophrenia showed highly modularized and significant differences in multiple network domains, whereas males and females showed less modular differences. Both cell count and average cluster size analysis for subgroups indicate a higher connectivity rate in the fourth quartile for the visual network in the control group. This indicates increased trSC in visual networks in the controls. In other words, this shows that the visual networks in people with schizophrenia have less mutually consistent spectra. It is also the case that the visual networks are less spectrally correlated on short timescales with networks of all other functional domains.

**Conclusions:**

The results of this study reveal significant differences in the degree to which spectral power profiles are coupled over time. Importantly, there are significant but distinct differences both between males and females and between people with schizophrenia and controls. We observed a more significant coupling rate in the visual network for the healthy controls and males in the upper quartile. Fluctuations over time are complex, and focusing on only time-resolved coupling among time-courses is likely to miss important information. Also, people with schizophrenia are known to have impairments in visual processing but the underlying reasons for the impairment are still unknown. Therefore, the trSC approach can be a useful tool to explore the reasons for the impairments.

## 1. Introduction

Over the last decade, noninvasive neuroimaging based on blood oxygenation-level dependent (BOLD) functional magnetic resonance (fMRI) has become a dominant technique for studying brain function in human subjects. There is now considerable evidence (Demirtas et al., 2016; Du et al., 2020; Xu et al., 2022) that aberrant functional connectivity plays a role in multiple neuropsychiatric disorders. Both the clinical specificity of such findings and their prospects for yielding actionable insights into disease mechanisms can be enhanced by studying new features of the fMRI signal. Various methods have been developed to study functional connectivity (FC). Early work on FC focused on so-called “static” connectivity analysis, which does not capture changes in the signals' correlative relationships through time (Friston, 1998; Saha et al., 2005). These changes over time are captured by a dynamic connectivity analysis method, which is typically evaluated on relatively short sliding windows through a more extended fMRI scan (Sakoglu et al., 2010; Hutchison et al., 2013; Calhoun et al., 2014; Damaraju et al., 2014). The results of these analysis have been highly informative on distinguishing the differences between healthy controls and patients. Most neuropsychiatric disorders can be distinguished from healthier brain conditions via connectivity analysis during the resting state. Resting-state dynamics, i.e., the functional brain dynamics in the absence of any stimulus or task, has become an important experimental protocol in clinical imaging research, and fMRI is a technology that has been widely used for brain mapping, connectivity, and for investigating resting-state dynamics in schizophrenia (Friston, 1998; Deco et al., 2013). While the link between mental disorders and connectivity is heavily studied within the clinical fMRI research community, brain-based underpinnings and the functional characteristics of schizophrenia remain unclear.

Besides the analysis in the time domain, several studies have shown that spectral analysis of the fMRI signals can also provide information about differences in connectivity (Miller et al., 2015; Agcaoglu et al., 2022), and recently few studies focused on the frequency domain of functional connectivity (Calhoun et al., 2012; Yaesoubi et al., 2017a; Tan et al., 2018). In addition to the studies suggesting significant spectral differences in the people with schizophrenia (Yaesoubi et al., 2017b; Li et al., 2022), more recent work has also shown that the differences in spectral manifest over time and across brain regions (Thompson and Fransson, 2015). Previous work has examined the correlation between whole-signal power spectra (Beer and Norton, 1988; Overath et al., 2008; Friston, 2011).

**Figure 1:**
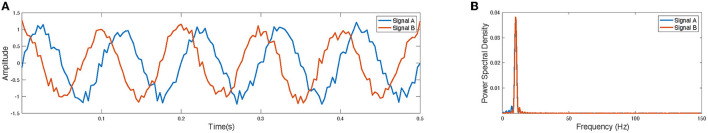
A toy example: **(A)** Two artificial-time courses with the same frequency and a phase shift of π/2. **(B)** The power density spectrum of the artificial signals.

In this study, we introduce a novel method that adapts the sliding-window technique to analyze the time-resolved spectral coupling between distinct brain networks. To illustrate our motivation and benefit of the proposed method better, we present a “toy" example, we generated two artificial signals representing random network time courses, signal A and signal B (Figure 1). Signals have the same frequencies and a phase shift of π/2. While these signals are poorly correlated in the time domain, they are highly correlated in the frequency domain which shows that the spectral analysis can also provide features which are neglected in the time domain analysis such as time courses with delay. We observed that the power spectra of brain circuits during the resting state are dynamic in time. We then investigated the pairwise correlation between the time-varying power spectra of brain circuits. These observations led us to develop a novel method of analyzing the spectral coupling in the subgroups of data composed of the resting-state fMRI recordings from people with schizophrenia (SZ) and healthy controls (HC). Our method is based on a time-resolved dynamic spectral connectivity analysis approach using the spectral correlation between windowed time-courses of paired brain circuits identified via independent component analysis (ICA).

The primary goal here is to develop a new method for analyzing the dynamics of resting-state fMRI scans and finding biomarkers for clinical groups for diagnosis and gender. Our results showed that the HCs exhibit robust connectivity in visual networks compared to SZ.

## 2. Methods and materials

**Figure 2:**
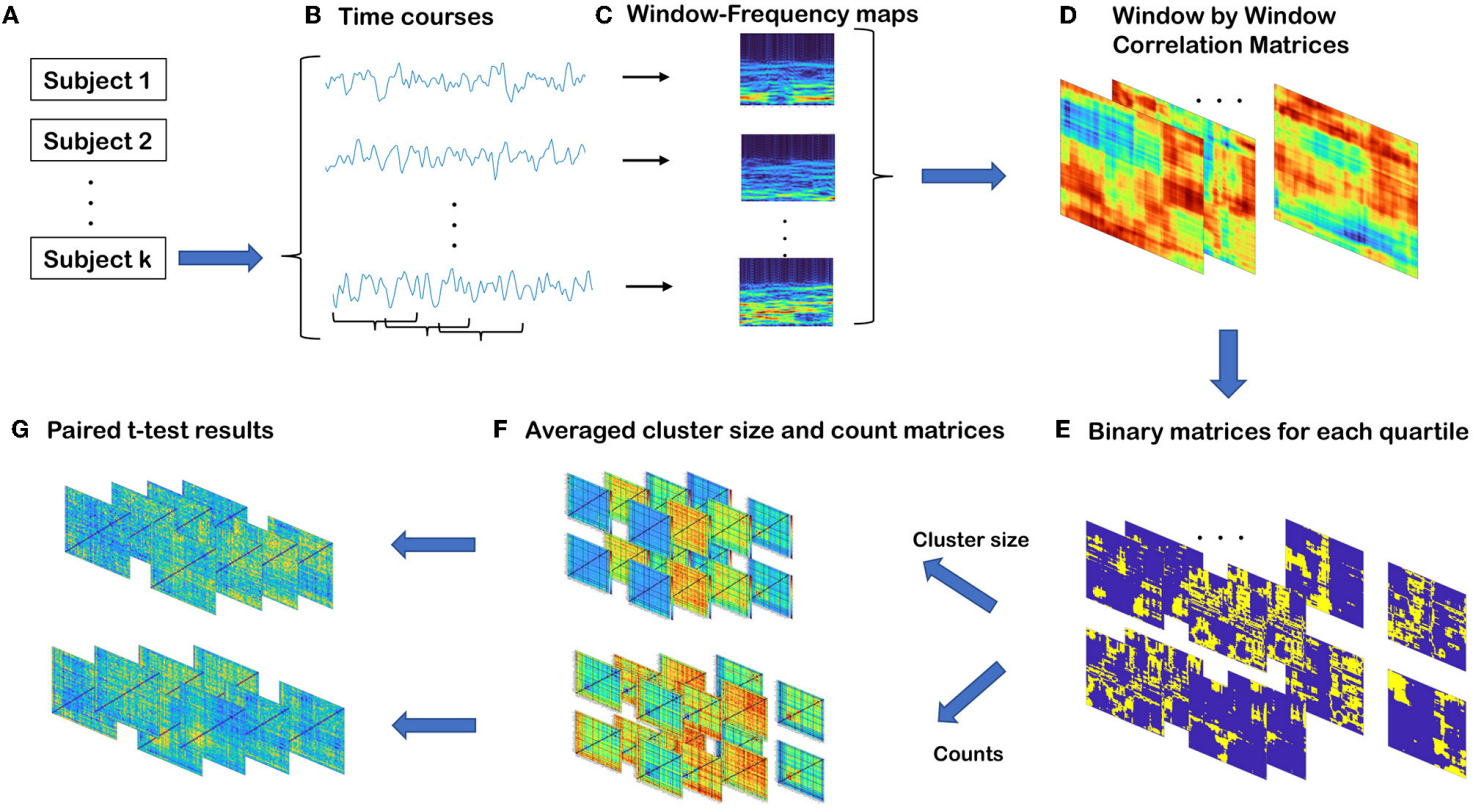
Summary of steps involved in time-resolved spectral coupling analysis. **(A)** Three hundred fourteen subjects in total 151 (114 males, 37 females) SZ and 163 (117 males, 46 females) HC. **(B)** For each subject, there are 47 components and each of them is associated with a windowed time-course. **(C)** Power spectra of windowed time-courses. The sizes of the matrices are *S* by *T* where *S* is the half of window length and *T* is the number of windows. **(D)** Correlation matrices of windowed power spectra for all possible component pairs. The sizes of the matrices are T by T. **(E)** By collapsing all WW matrices for all subjects, global quartile values are computed. By using these quartile values, four binary matrices are defined for each WW matrix. The sizes of each matrix are the same as WW matrices. **(F)** Average cluster size and the average count of cells in each quartile. **(G)** Regression analysis results for each averaged count and average cluster size matrices in each quartile to examine the group differences.

As illustrated in Figure 2, we developed a novel method for capturing transient spectral coupling based on a sliding window approach. First, we evaluated time-resolved spectral coupling (trSC) by calculating the correlation between power spectra of windowed time course pairs of brain circuits. We then estimated the spectral coupling as described below and, finally, evaluated differences in these measures in the schizophrenia subjects vs. healthy controls and between males and females. The steps are explained in detail in the following section.

### 2.1. Participants and preprocessing

**Figure 3:**
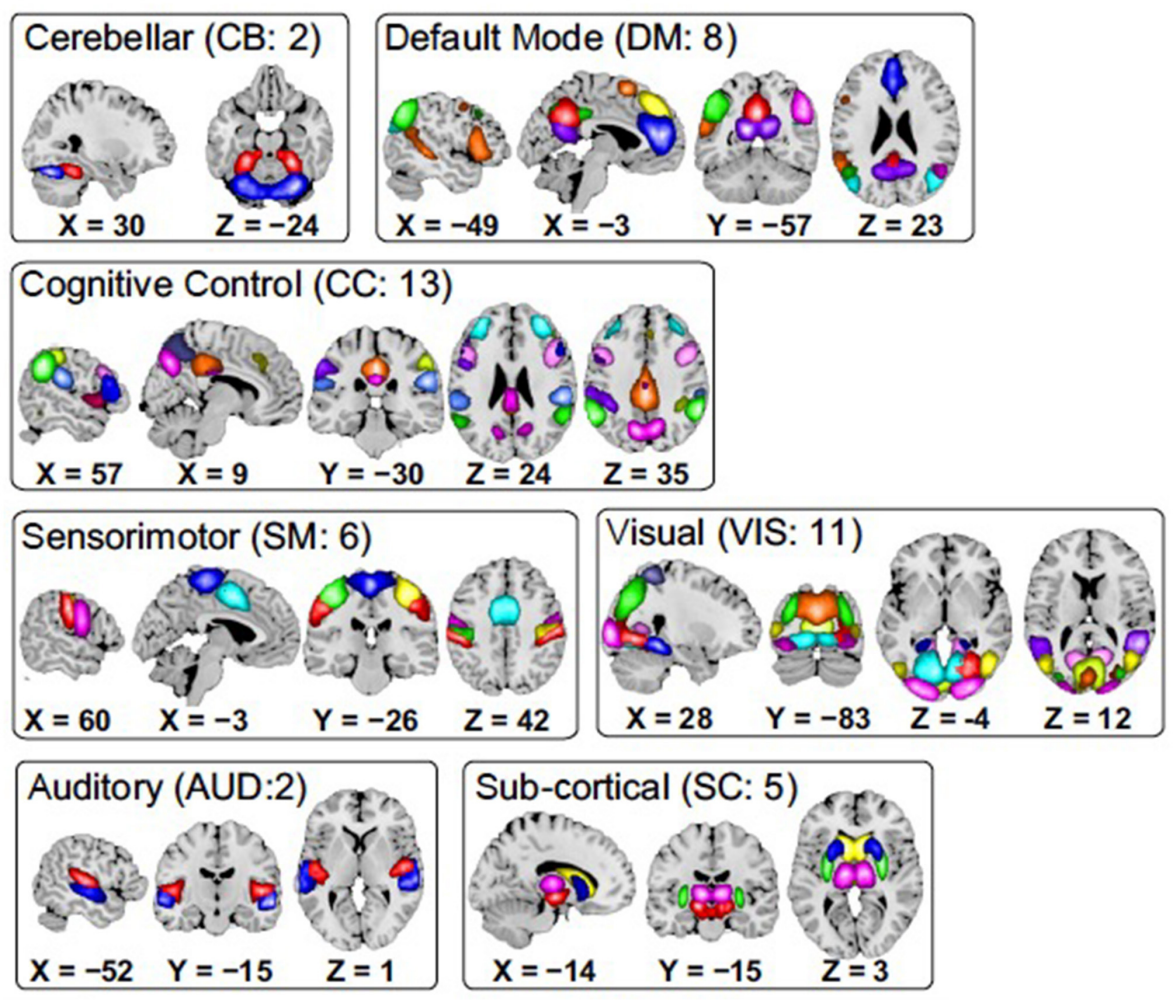
Spatial maps of the 47 identified brain components, grouped into seven subcategories. Each color in the maps within each domain corresponds to a unique component.

Data used in this work is a collection of resting fMRI data collected from 151 (114 males, 37 females; mean age 37.8) schizophrenia subjects (SZ) and 163 (117 males, 46 females; mean age 36.9) healthy controls (HC), which is a part of the Functional Imaging Biomedical Informatics Research Network (FBIRN) project (Potkin and Ford, 2008). The participants were selected from seven different locations in the United States. Acquisition and preprocessing are explained in detail in Damaraju et al. (2014). Resting fMRI data were collected at eyes closed condition using 3 Tesla scanners with a resolution of 3.4375 × 3.4375 × 4 (including a 1 mm gap) mm^3^, a repeat time (TR) of 2 s, and an echo time (TE) of 30 ms, over a five-minute period, resulting in 159 timepoints. Briefly, fMRI data were processed through an SPM pipeline, including slice-timing and motion correction, spatial normalization to the Montreal Neurological Institute (MNI) template, resampling to 3 × 3 × 3 mm^3^ data, and spatial smoothing with a 6 × 6 × 6 mm^3^ full width at half maximum (FWHM) Gaussian kernel. Group independent component analysis (gICA) (Du et al., 2020) was used to estimate 100 circuits, out of which 47 distinct intrinsic networks were identified and ordered in a previous study (Damaraju et al., 2014). Single subject maps and time-courses were back-reconstructed. Each component is associated with a time course for each subject. These 47 components were grouped into seven distinct networks: subcortical (SC), auditory (AUD), visual (VIS), sensorimotor (SM), cognitive control (CC), default mode network (DM), and cerebellar (CB). These distinct networks are illustrated in Figure 3.

### 2.2. Dynamic spectral connectivity

To evaluate changes over time in the spectral coupling, we use a sliding window approach. The time courses were split into overlapping windows of length 50 TRs, and windows were shifted 1 TR over the entire time-course. The time domain includes 159 samples (2 s each) for each time course, resulting in 109 overlapping windows for each component. All analyses were performed using MATLAB (MAT, 2021). We first computed a spectrogram for each time course via a windowed Fourier transform (FFT). The fluctuations over the frequencies were observed via the spectrograms through the variations of windows in each time course. The spectogram calculation resulted in 47 window-by-frequency matrices for each of 314 subjects. Next, we computed window by window (WW) spectral coupling of the power spectra for each pair of components throughout all subjects. We obtained 1,081 WW correlation maps for each subject during this computation. These WW maps were then averaged over subgroups to observe the coupling properties.

### 2.3. Clustering

We presented our results using two summary measures, cluster size and cell counts, for each quartile. In addition, global quartile values were computed using WW correlation maps throughout all subjects and all possible component pairs. Using the global quartiles of the system, we calculated the number of cells in each quartile for each WW map. Four binary matrices for the count of cells in each quartile are computed for each WW correlation map. These maps were averaged over the subgroups. Also, using binary quartile matrices, we calculated the average cluster size within the various subgroup of interest. Clusters are composed of vertically and horizontally adjacent cells within the binary quartile matrices. In order to keep the analysis distinctive, we did not consider the diagonally adjacent cells as part of the clusters. We calculated the cluster size for each quartile of the WW matrices, followed by a similar cell count analysis protocol, and averaged them over the subgroups. We summarize the findings in the results section.

### 2.4. Statistical analysis

We performed regression analysis on average cluster size and cell counts between healthy control vs. patient and male vs. female groups in each quartile to examine the group differences. The significance level was set to 0.05 for *p*-value, corrected using Benjamini–Hochberg method for multiple comparisons using a false discovery rate (FDR) approach (Benjamini and Hochberg, 1995).

## 3. Results

Following previous studies of functional brain connectivity, time courses were computed through the independent component analysis (Salman et al., 2019). We developed a new method with two measures, cell-count, and average cluster size, to investigate the dynamic spectral coupling strength for healthy controls, schizophrenia subjects, males, and females within each quartile. Both measures showed a higher connectivity rate for the visual network in the last quartile for the males and the control groups. This indicates that trSCs are more robust in the visual network for controls or a less correlated visual network for schizophrenia subjects compared to the control group. Also, we performed a multiple regression analysis for both measures to observe statistical differences between subgroups. Here, we present our detailed results for the cluster size and cell-count analyses in this section and for the analysis results for lower quartiles; see Supplementary material 1.1.1.

### 3.1. Cluster size

**Figure 4:**
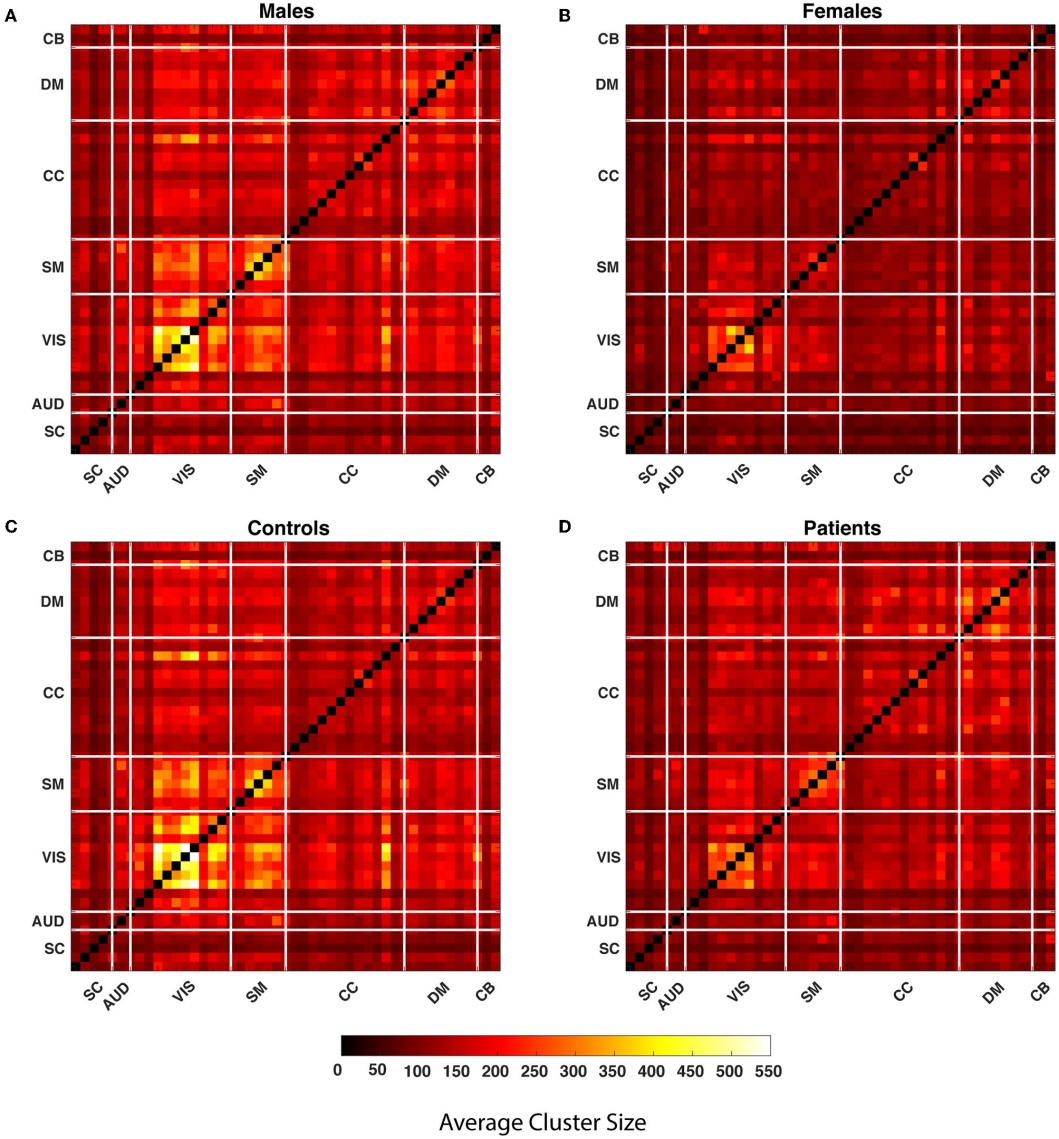
The average cluster size is calculated throughout all WW correlation maps for each quartile within the subgroups. In the figure, each cell of the matrices represents the average cluster size of a particular component pair for the subgroups. The matrices represent the average cluster size of the male, female, healthy controls and people with schizophrenia in the last quartile from **(A–D)**. The thick white lines split the map into regions for seven distinct networks defined in the methods section.

Our observations show that the comparison of the HC-SZ and male-female show the greatest differences in the upper quartile, so we primarily highlight aspects of the upper quartile. A 1,081, 109 by 109, WW correlation maps are computed for each pair of 47 components. Global quartiles are calculated for the vector formed by collapsing all WW maps and lastly, four binary matrices for each WW correlation map are calculated for each quartile. The clusters in each quartile are composed of vertically and horizontally adjacent cells. While defining the clusters, discrete single cells are also considered as clusters. We calculated the average cluster size for all WW correlation maps in every quartile and averaged them over each pair of components for males, females, SZ subjects, and healthy controls. Figure 4 shows the average cluster size for the subgroups. The maps show that the average cluster size is significantly higher for the visual network in males and the control group compared to females and SZ subjects.

**Figure 5:**
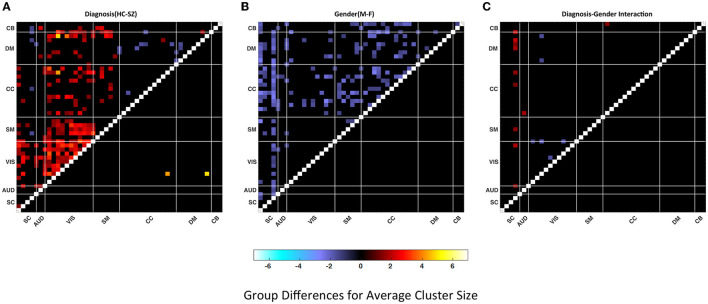
Group differences in **(A)** Healthy control—patient, **(B)** Male–female, **(C)** Diagnosis-gender interactions for average cluster size. Values are plotted for thresholded *p*-values* -log_1_0 (*p*-value) sign (t-statistic) where *p*-values masked for significance at *p* ≤ 0.05 in the upper triangular matrices and thresholded FDR corrected *p*-values* –log_1_0 (*p*-value) sign (t-statistic) where FDR corrected *p*-values are masked for significance via Benjamini–Hochberg procedure in the lower triangular matrices. Statistics are obtained via linear regression analysis (red shades represent the dominance of HC or F and the blue shades represent the dominance of SZ or M).

In Figure 5, we present the group difference matrices for female-male, SZ-HC groups and their interactions. The upper triangles of all three matrices represent the p-values thresholded at *p* < 0.05 multiplied by −log_10_(*p*)*sign*(*t*) via regression analysis. The lower triangle of the matrices represents FDR corrected via Benjamini–Hochberg method multiplied by −log_10_(*p*)*sign*(*t*). The statistics are obtained via multiple regression analysis. There are just two significant pairs for the HC-SZ comparison while there are none for gender and interaction cases. Similar outcomes can be observed for the lower quartiles.

### 3.2. Cell counts

**Figure 6:**
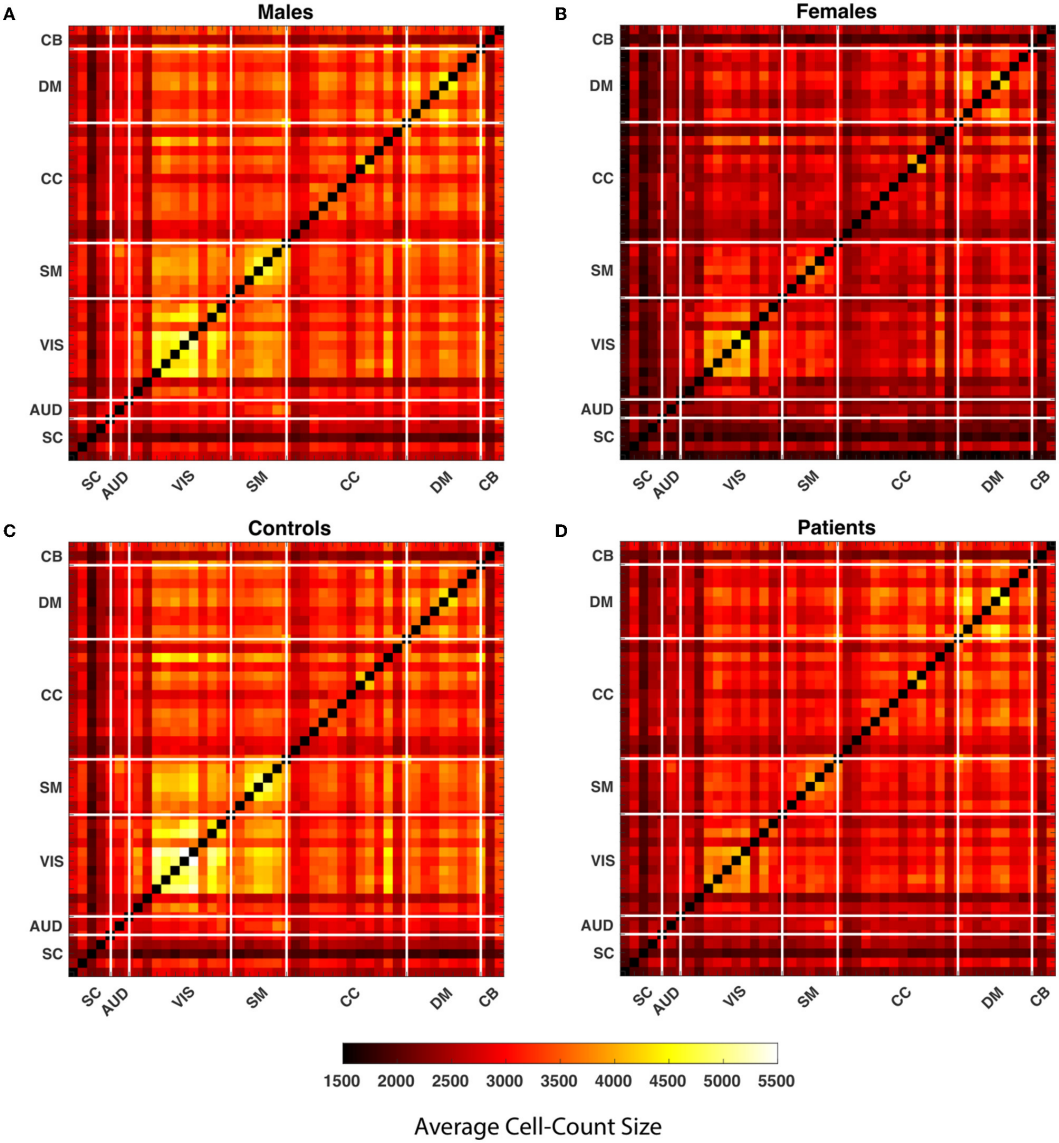
The average cell count is calculated throughout all WW correlation maps for each quartile within the subgroups. In the figure, each cell of the matrices represents the average cell count of a particular component pair for the subgroups. The matrices represent the average cell count of the male, female, healthy controls and people with schizophrenia in quartile four from **(A–D)**. The thick white lines split the map into regions for seven distinct networks defined in the methods section.

**Figure 7:**
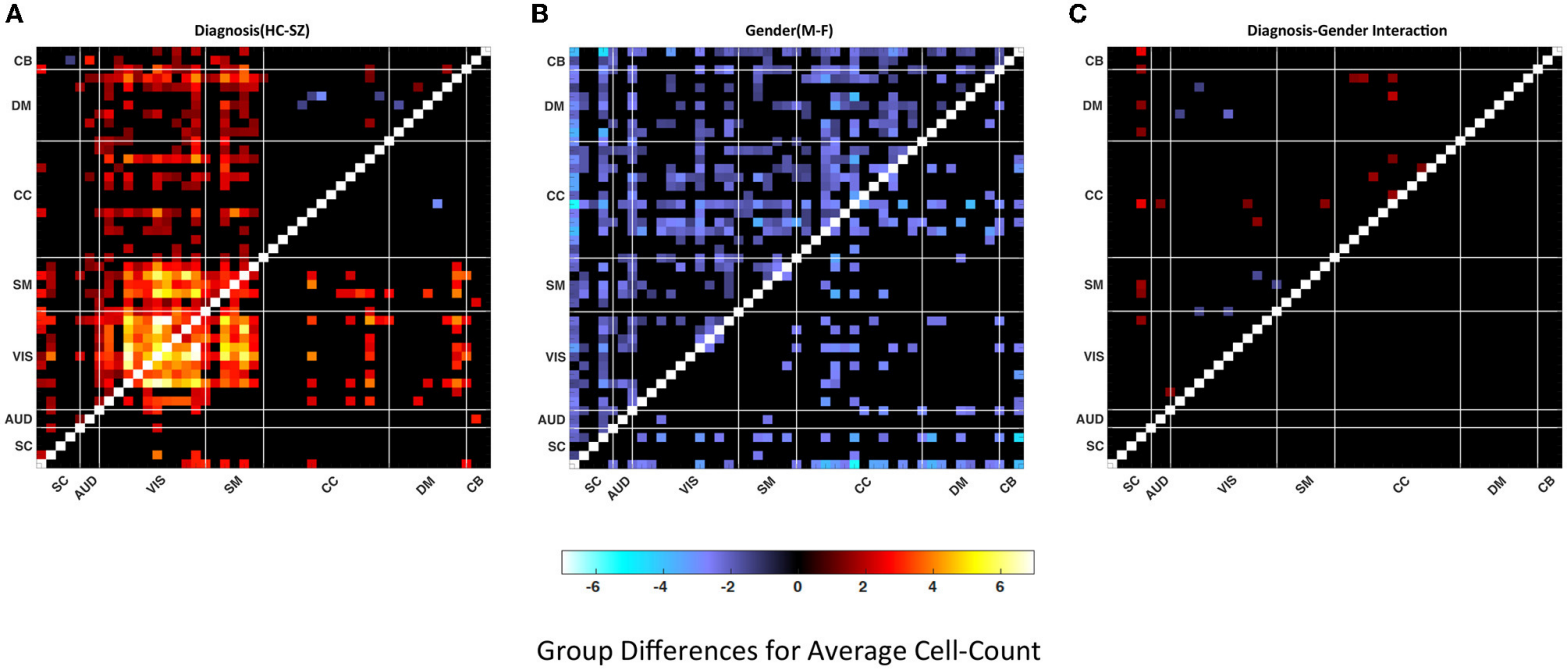
Group differences in **(A)** Healthy control—patient, **(B)** Male–female, **(C)** Diagnosis-gender interactions for average cell-count. Values are plotted for thresholded *p*-values*–log_1_0(*p*-value) sign (t-statistic) where *p*-values masked for significance at *p* ≤ 0.05 in the upper triangular matrices and thresholded FDR corrected *p*-values* –log_1_0 (*p*-value) sign (t-statistic) where FDR corrected *p*-values are masked for significance via Benjamini–Hochberg procedure in the lower triangular matrices. Statistics are obtained via linear regression analysis (red shades represent the dominance of HC or F and the blue shades represent the dominance of SZ or M).

Both measures used the same set of quartiles for the analysis. Figure 6 illustrates average cell-counts for the subgroups. The number of cells, or cell counts, was calculated for the binary matrices in every quartile and averaged over subgroups. Here, like the cluster size analysis, our results are focused on the aspects of the last quartile. Compared to the first three quartiles, we observed more significant results in the last quartiles. We also observed that in the lower quartiles the dominance of the groups reverses. In other words, in the lower quartiles the people with schizophrenia and female are more dominant while there is no significance for the interactions; see Supplementary material 1.1.1. As seen in Figure 6, comparing SZ and HC groups, we observed that the HC group has higher cell counts or trSC in the visual network, similar to the cluster size analysis. In addition to the visual network, we also observed higher coupling strength in the sensorimotor. We again analyzed the group differences for the female–male and SZ-HC via a regression analysis with a significance level *p* < 0.05 which is presented in the upper triangular parts of the matrices and FDR corrected results are presented in the lower triangular parts of matrices in Figure 7.

The results demonstrate a higher connectivity rate for the visual network in the HC case in the fourth quartile compared to other quartiles and SZ subjects. We also observe significant differences in the gender comparison however they are much more widespread across multiple brain networks and do not have the same characteristics as the diagnosis contrast. The increased connectivity rate in the last quartile implies that high-frequency spectral fluctuations are associated with greater correlation in the visual network.

## 4. Discussion

Information is conducted through the brain regions in various ways. Frequency differences in communication of brain networks based on function and on mental health seem likely but have not been well-studied (Daitch et al., 2013). This study investigated the differences in time-resolved spectral coupling for resting-state fMRI between controls and schizophrenia subjects and between genders. Our results contribute to the limited literature on functional spectral coupling in schizophrenia. Previous studies (Damaraju et al., 2014; Sendi et al., 2021) have shown that the visual network has a higher correlation rate for HC compared to the SZ. Here, the results are extending certain earlier findings arising from different methods to a time-resolved spectral coupling for resting-state fMRI between controls and schizophrenia subjects and between genders.

Another method proposed in earlier studies for a similar purpose is coherence (Chang and Glover, 2010; Yang et al., 2018; Hsu et al., 2022). The purpose of coherence and correlation is similar, but they diverge in detail. One advantage of trSC method is its narrower sensitivity to linear frequency coupling over the full spectrum and the lack of any underlying assumption about stationarity. Coherence will identify strong frequency-specific spectral coupling but averaging across frequencies — the most natural scalar summary measure characterizing full spectrum coherence between two time-series—can blur the shape of the relationship between the spectra; see Supplementary material 1.1.2. To ensure that the measure is specifically identifying linear common patterning in the (windowed) power spectral densities (PSDs) of two time-series, the correlation is a more direct approach, less likely to blur different ways that windowed PSDs might exhibit co-spectral properties.

While not exactly the same as what we present here, correlation is a commonly used connectivity measure in frequency studies with combination of other studies such as PSD (Li et al., 2022) and amplitude of low-frequency fluctuations (ALFF) and full spectral comparisons (Calhoun et al., 2012; Di et al., 2013; Fu et al., 2018; Wang et al., 2019).

Our primary aim was to develop a method for analyzing the spectral coupling between brain components and networks. Our results are focused on the upper quartile, where the coupling rate or connectivity level is higher relative to the lower quartiles. The cell-count analysis showed a higher connectivity level in the visual network observed among males and among controls compared to females and schizophrenia subjects whereas the cluster size analysis showed much fewer differences for the group comparisons. The lower connectivity level in the visual networks among people with schizophrenia for the fourth quartile indicates decreased trSC, i.e., less mutually consistent spectra, in visual networks among people with schizophrenia. It is also the case that the visual networks are less spectrally correlated in SZs on short timescales with non-visual functional domains.

The visual network in schizophrenia is not an intensively-investigated area in resting-state fMRI research. Still, it is known that people with schizophrenia have visual processing impairments, and visual networks are likely to play a significant role in the disorder (Sendi et al., 2021). It also is known clinically that all is not well with visual processing in schizophrenia, which in turn results in impaired social cognition and social deficits (Javitt, 2009). There is hyperawareness for selective visual stimuli at the price of selective suppression of others — usually misclassifying background noise as significant. Visual mis-processing seems to be one component leading to impaired social cognition and social deficits and all the way to paranoid delusions (Taylor et al., 2011).

The cell-count analysis demonstrated significant differences in visual networks between HC and SZ, while the significant difference between the gender groups is scattered along the component pairs with male dominance. In addition to the group differences in the visual network trSC, we also observed significant differences in the sensorimotor network in cell-count analysis (Cadenhead et al., 2013; Yaesoubi et al., 2017a). The cluster size analysis did not provide the same results in the VIS and SM networks. Also, in both analyses, there is no significant difference in the interaction of diagnosis and gender. A possible interpretation of visual network-related coupling differences is more elaborated between patients, and control can be related to data being collected in eyes closed conditions. Eyes closed condition is a less controlled condition compared to eyes open studies and more sustainable to drowsiness and mind wandering, hallucination which may affect people with schizophrenia more dramatically compared to the control, but further studies are needed to conclude this (Agcaoglu et al., 2019; Weng et al., 2020).

Another interesting outcome of our results is that while in all other networks, both cluster size and cell count are higher in control compared to patients, DM-DM and DM-CC coupling are higher in patients. DM is a well-studied network in schizophrenia research, and schizophrenia is usually associated with altered activity, and altered connectivity of DM (Guo et al., 2017).

## 5. Limitations

We should mention some limitations of the method. In this study, the window length is 50 TR and the time shift is 1 TR. Variations in the window length and the time shift can be decided according to the purpose of the study. E.g., smaller window length would be more sensitive to more rapid changes over time. For small variations in the window length, we did not observe dramatic changes in averaged cluster size and cell count measures; see Supplementary material 1.2. Since our focus in this study is not the window length, we chose a window length relatively large. Ultimately, the window size is a filtering choice, prior work (Faghiri et al., 2020) provides a unified framework to evaluate filters across the entire available range, which we plan to investigate in a future study.

## 6. Conclusion

The brain networks communicate with each other in different ways. In this study, we proposed a frequency domain method to measure this network communication and we found significant differences in the degree to which spectral power profiles are coupled over time. The results showed significant but distinct differences between males vs. females and schizophrenia subjects vs. controls. The results might suggest that the information is conducted through the brain via different spectral scales. The results also showed reduced visual/sensorimotor coupling in SZ vs. HC and also reduced multi-network subcortical coupling in females vs. males. Fluctuations over time are complex, and focusing on only time-resolved coupling among time-courses is likely to miss important information. The trSC approach should be further studied in future work.

## Data availability statement

The data analyzed in this study is subject to the following licenses/restrictions. The FBIRN dataset can be made available upon a reasonable request made to the corresponding author and contingent upon IRB approval. Requests to access these datasets should be directed at: info@trendscenter.org.

## Author contributions

DA: conceptualization, methodology, investigation, formal analysis, writing original draft and review, software, and visualization. RM: conceptualization, methodology, and review. OA: methodology, data visualization, and review. AP and JF: data curation. VC: supervision, conceptualization, methodology, and review. All authors contributed to manuscript revision, read, and approved the submitted version.
