# Supplementary material for: A method for capturing dynamic spectral coupling in resting fMRI reveals domain-specific patterns in schizophrenia
Source: Front Neurosci. 2023 Apr 27;17:1078995. doi: 10.3389/fnins.2023.1078995 (PMC10174238; doi:10.3389/fnins.2023.1078995)
Supplement: Supplementary file 1 [file Data_Sheet_1.PDF]

Supplementary Material

1 SUPPLEMENTARY TABLES AND FIGURES

1.1 Figures

1.1.1 Group Differences for Q1,Q2,Q3

Cluster size and cell count analysis results for the lower quartiles. Since the group differences in the lower quartiles are less significant compared to the upper quartile our results are focused on it.

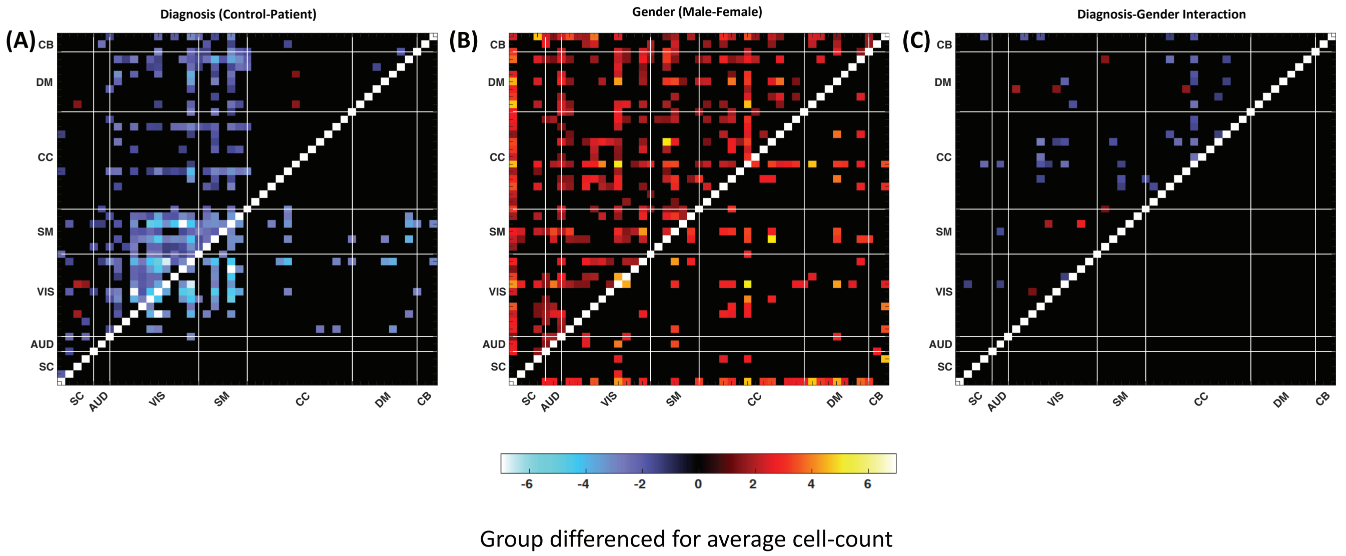

Figure S1. Group differences in (A) Healthy Control - Patient, (B) Male-Female (C) Diagnosis- Gender Interactions for average cell-count in the first quartile. Values are plotted for thresholded p-values*-log10(p-value) sign(t-statistic) where p-values masked for significance at $p \leq 0.05$ in the upper triangular matrices and thresholded FDR corrected p-values* -log10(p-value) sign(t-statistic) where FDR corrected p-values are masked for significance via Benjamini-Hochberg procedure in the lower triangular matrices. Statistics are obtained via linear regression analysis. (Red shades represent the dominance of HC or F and the blue shades represent the dominance of SZ or M.)

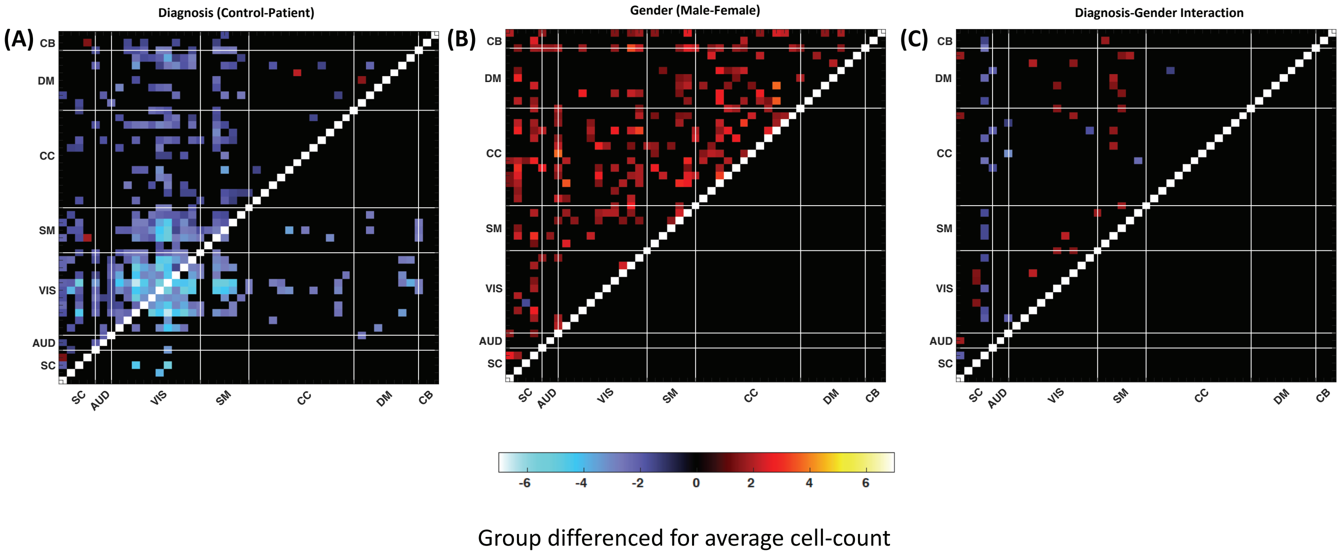

Figure S2. Group differences in (A) Healthy Control - Patient, (B) Male-Female (C) Diagnosis- Gender Interactions for average cell-count in the second quartile. Values are plotted for thresholded p-values*-log10(p-value) sign(t-statistic) where p-values masked for significance at $p \leq 0.05$ in the upper triangular matrices and thresholded FDR corrected p-values* -log10(p-value) sign(t-statistic) where FDR corrected p-values are masked for significance via Benjamini-Hochberg procedure in the lower triangular matrices. Statistics are obtained via linear regression analysis. (Red shades represent the dominance of HC or F and the blue shades represent the dominance of SZ or M.)

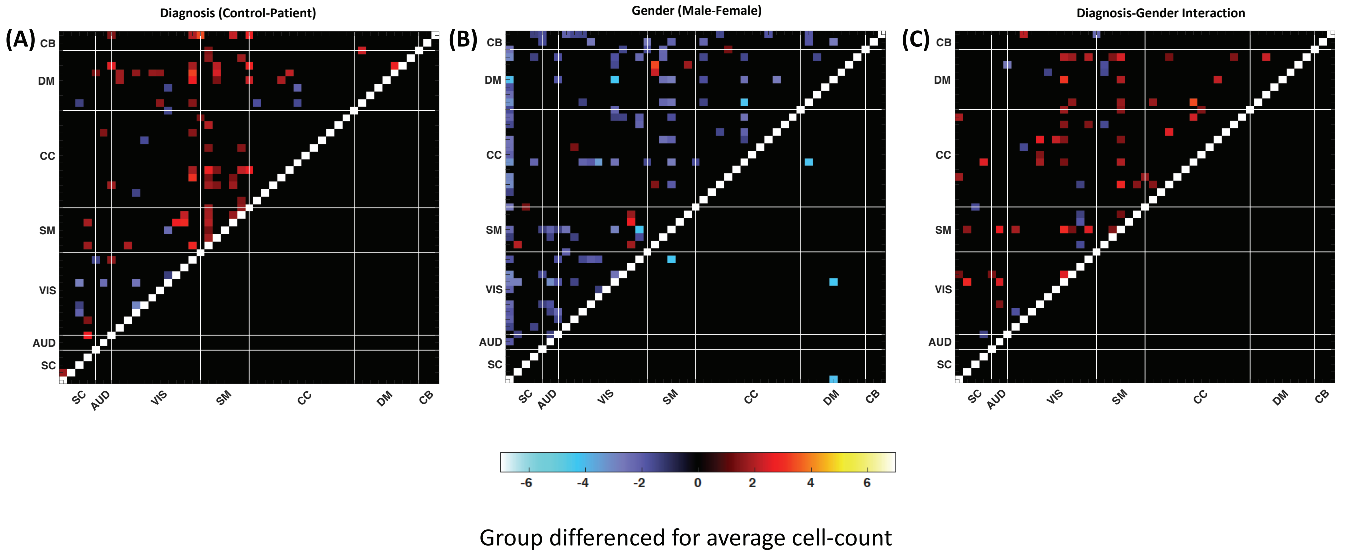

Figure S3. Group differences in (A) Healthy Control - Patient, (B) Male-Female (C) Diagnosis- Gender Interactions for average cell-count in the third quartile. Values are plotted for thresholded p-values*-log10(p-value) sign(t-statistic) where p-values masked for significance at $p \leq 0.05$ in the upper triangular matrices and thresholded FDR corrected p-values* -log10(p-value) sign(t-statistic) where FDR corrected p-values are masked for significance via Benjamini-Hochberg procedure in the lower triangular matrices. Statistics are obtained via linear regression analysis. (Red shades represent the dominance of HC or F and the blue shades represent the dominance of SZ or M.)

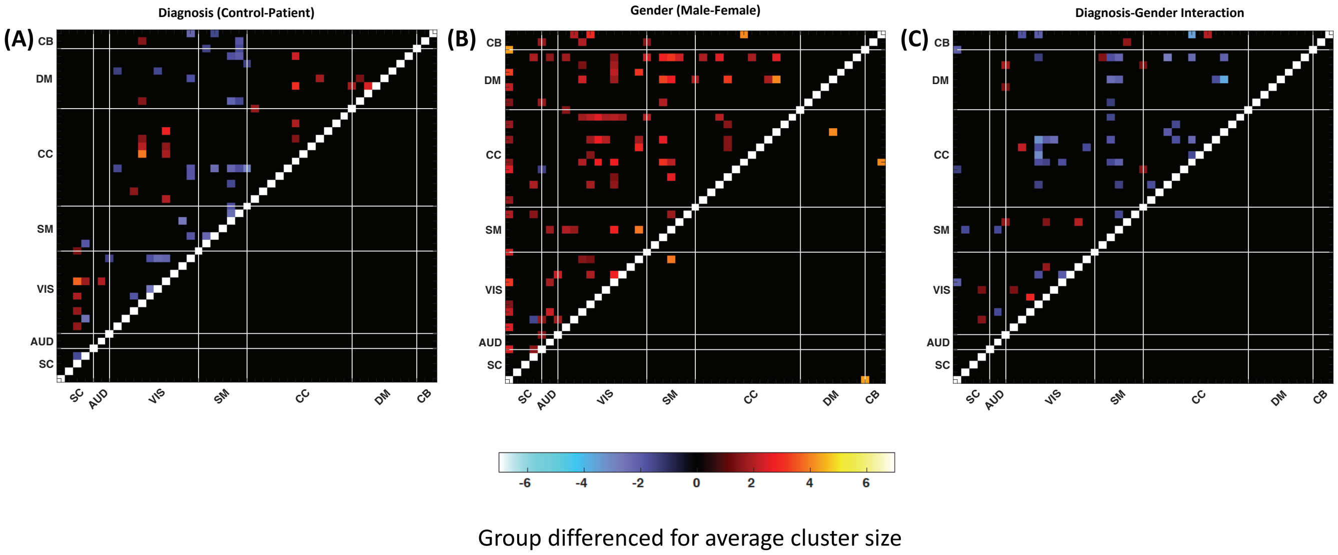

Figure S4. Group differences in (A) Healthy Control - Patient, (B) Male-Female (C) Diagnosis- Gender Interactions for average cluster size in the first quartile. Values are plotted for thresholded p-values*-log10(p-value) sign(t-statistic) where p-values masked for significance at $p \leq 0.05$ in the upper triangular matrices and thresholded FDR corrected p-values* -log10(p-value) sign(t-statistic) where FDR corrected p-values are masked for significance via Benjamini-Hochberg procedure in the lower triangular matrices. Statistics are obtained via linear regression analysis. (Red shades represent the dominance of HC or F and the blue shades represent the dominance of SZ or M.)

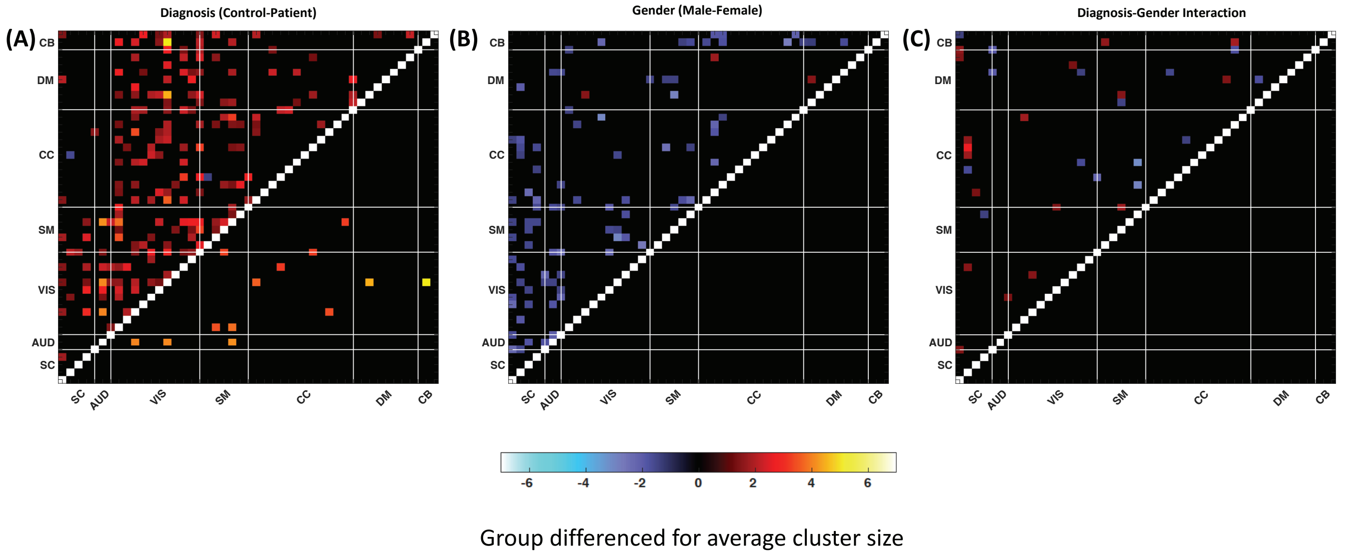

Figure S5. Group differences in (A) Healthy Control - Patient, (B) Male-Female (C) Diagnosis- Gender Interactions for average cluster size in the second quartile. Values are plotted for thresholded p-values*-log10(p-value) sign(t-statistic) where p-values masked for significance at $p \leq 0.05$ in the upper triangular matrices and thresholded FDR corrected p-values* -log10(p-value) sign(t-statistic) where FDR corrected p-values are masked for significance via Benjamini-Hochberg procedure in the lower triangular matrices. Statistics are obtained via linear regression analysis. (Red shades represent the dominance of HC or F and the blue shades represent the dominance of SZ or M.)

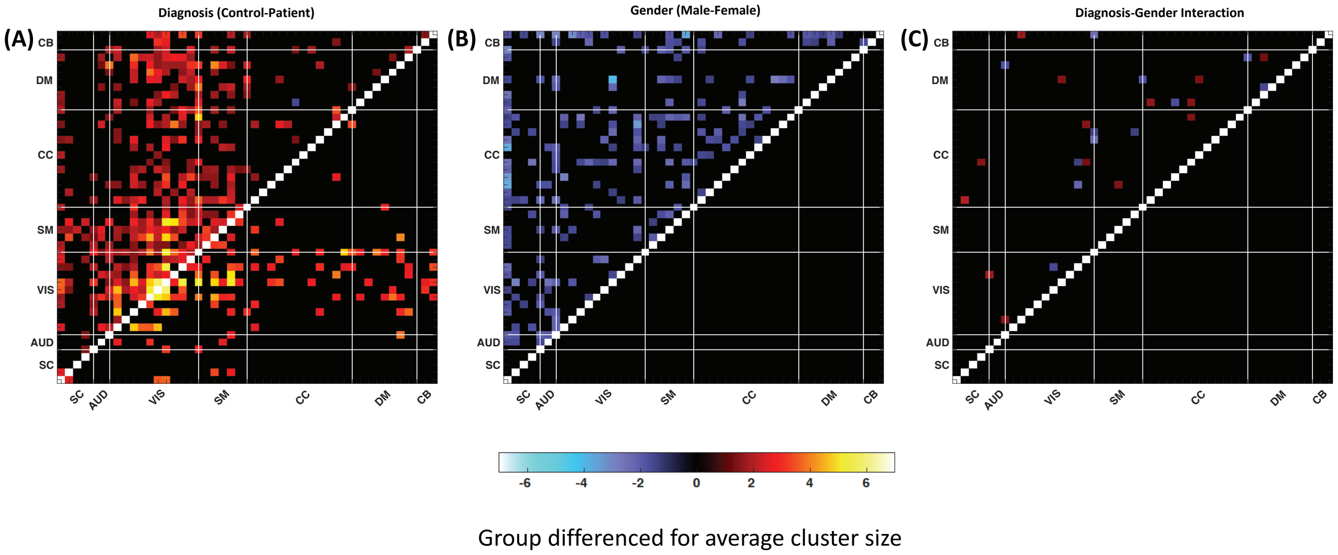

Figure S6. Group differences in (A) Healthy Control - Patient, (B) Male-Female (C) Diagnosis- Gender Interactions for average cluster size in the third quartile. Values are plotted for thresholded p-values*-log10(p-value) sign(t-statistic) where p-values masked for significance at $p \leq 0.05$ in the upper triangular matrices and thresholded FDR corrected p-values* -log10(p-value) sign(t-statistic) where FDR corrected p-values are masked for significance via Benjamini-Hochberg procedure in the lower triangular matrices. Statistics are obtained via linear regression analysis. (Red shades represent the dominance of HC or F and the blue shades represent the dominance of SZ or M.)

1.1.2 Coherence

Coherence analysis is performed for patient, control, and gender subgroups. As the figure presents, the difference between the subgroups is less significant compared to the proposed method.

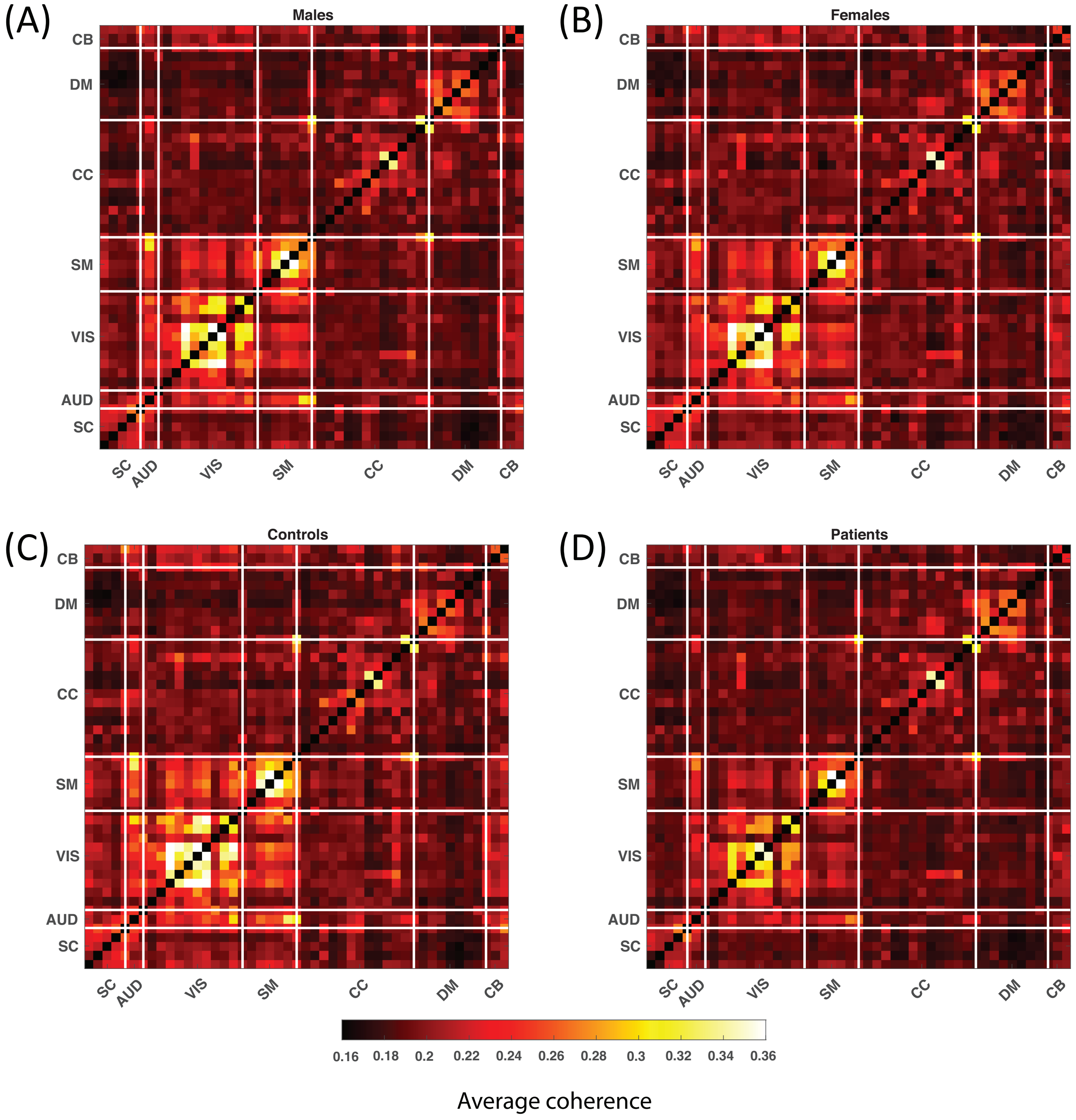

Figure S7. Coherence analysis results for subgroups (A) Male (B)Female (C) Control (D)Patient

1.2 Window Size Effect

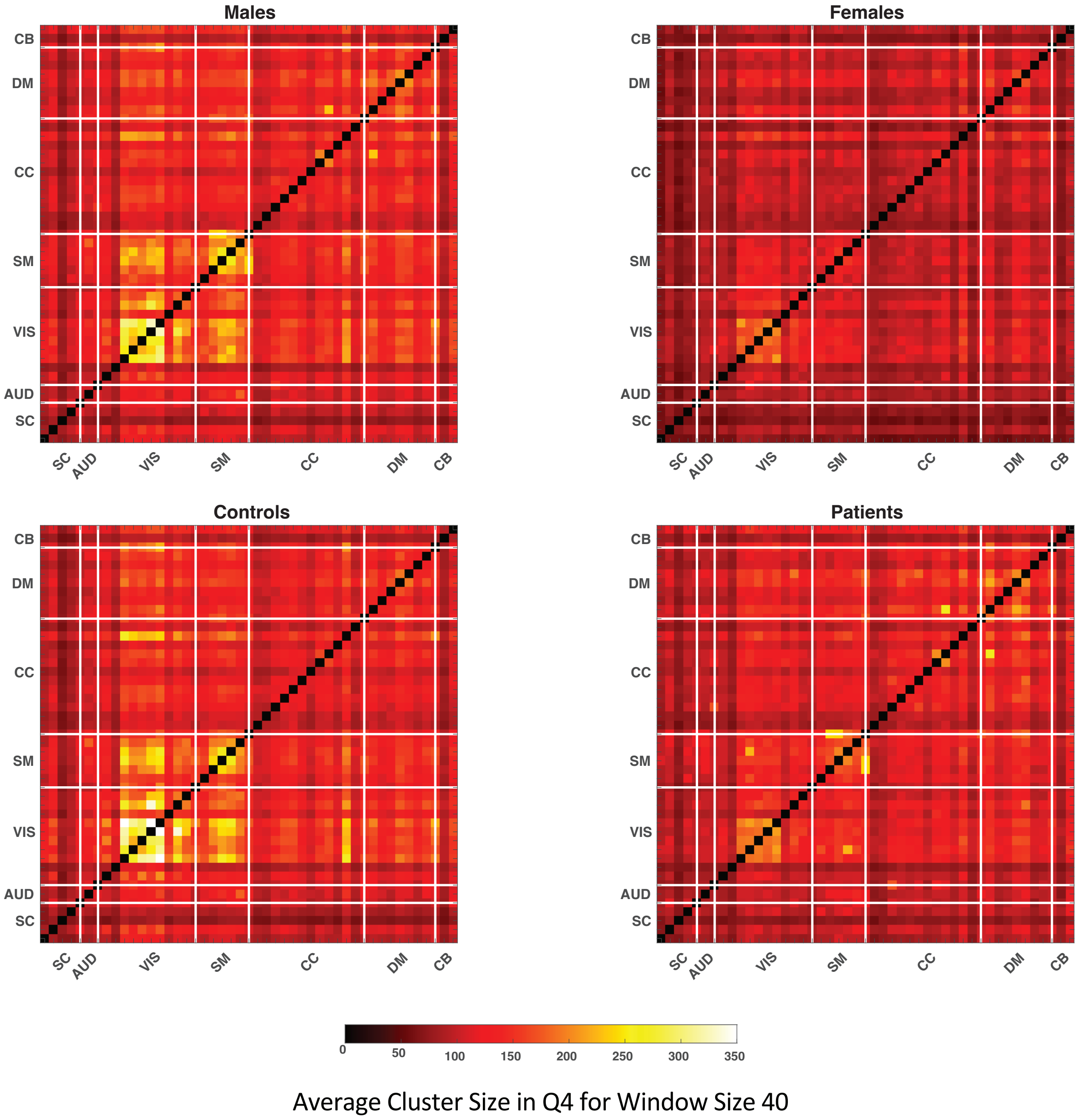

Figure S8. The average cluster size is calculated throughout all WW correlation maps for each quartile within the subgroups. In the figure, each cell of the matrices represents the average cluster size of a particular component pair for the subgroups. The matrices represent the average cluster size of the male, female, healthy controls and people with schizophrenia in the last quartile from A to D with window size 40TR. The thick white lines split the map into regions for seven distinct networks defined in the methods section.

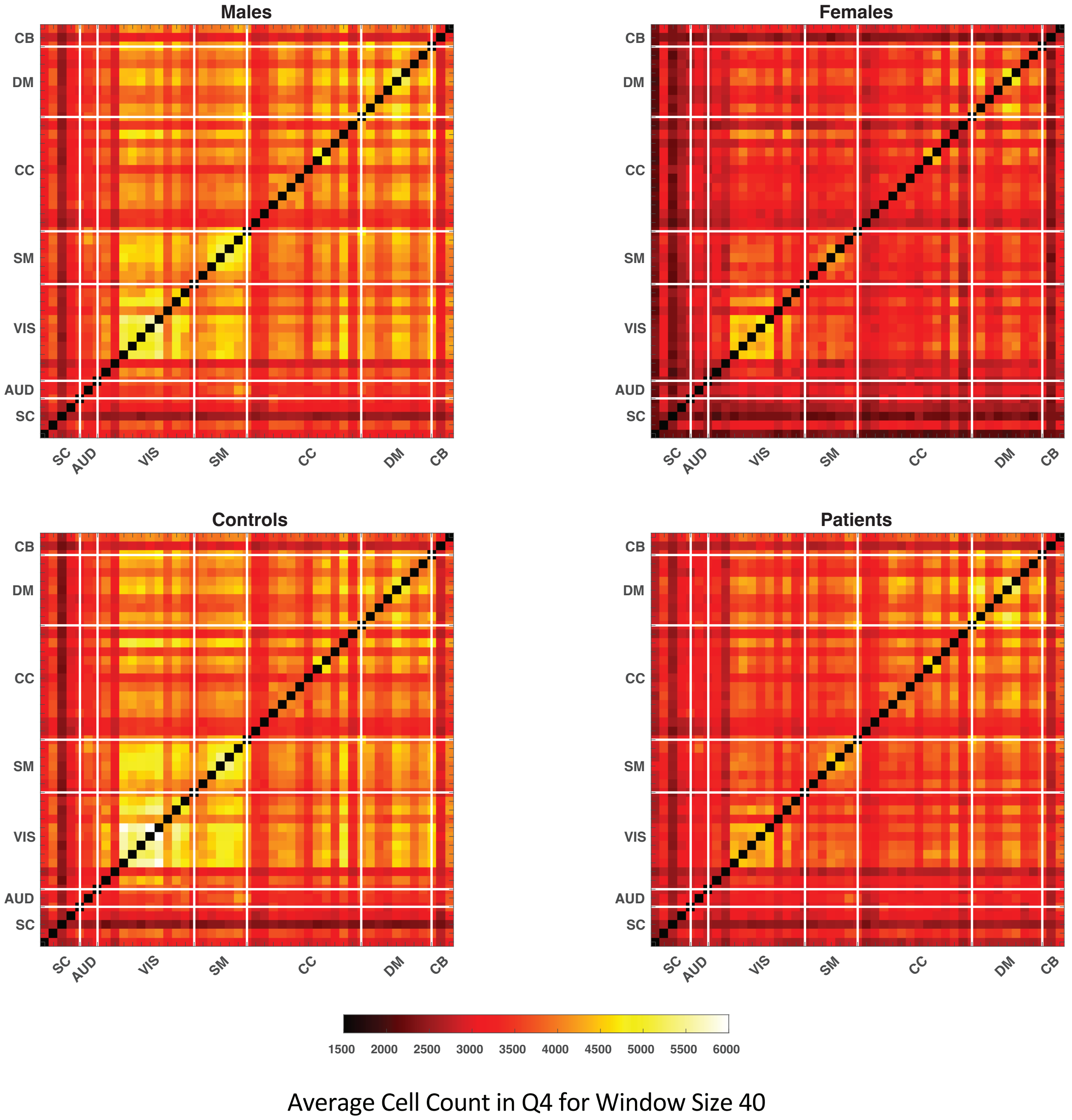

Figure S9. The average cluster size is calculated throughout all WW correlation maps for each quartile within the subgroups. In the figure, each cell of the matrices represents the average cell-count of a particular component pair for the subgroups. The matrices represent the average cluster size of the male, female, healthy controls and people with schizophrenia in the last quartile from A to D with window size 40TR. The thick white lines split the map into regions for seven distinct networks defined in the methods section

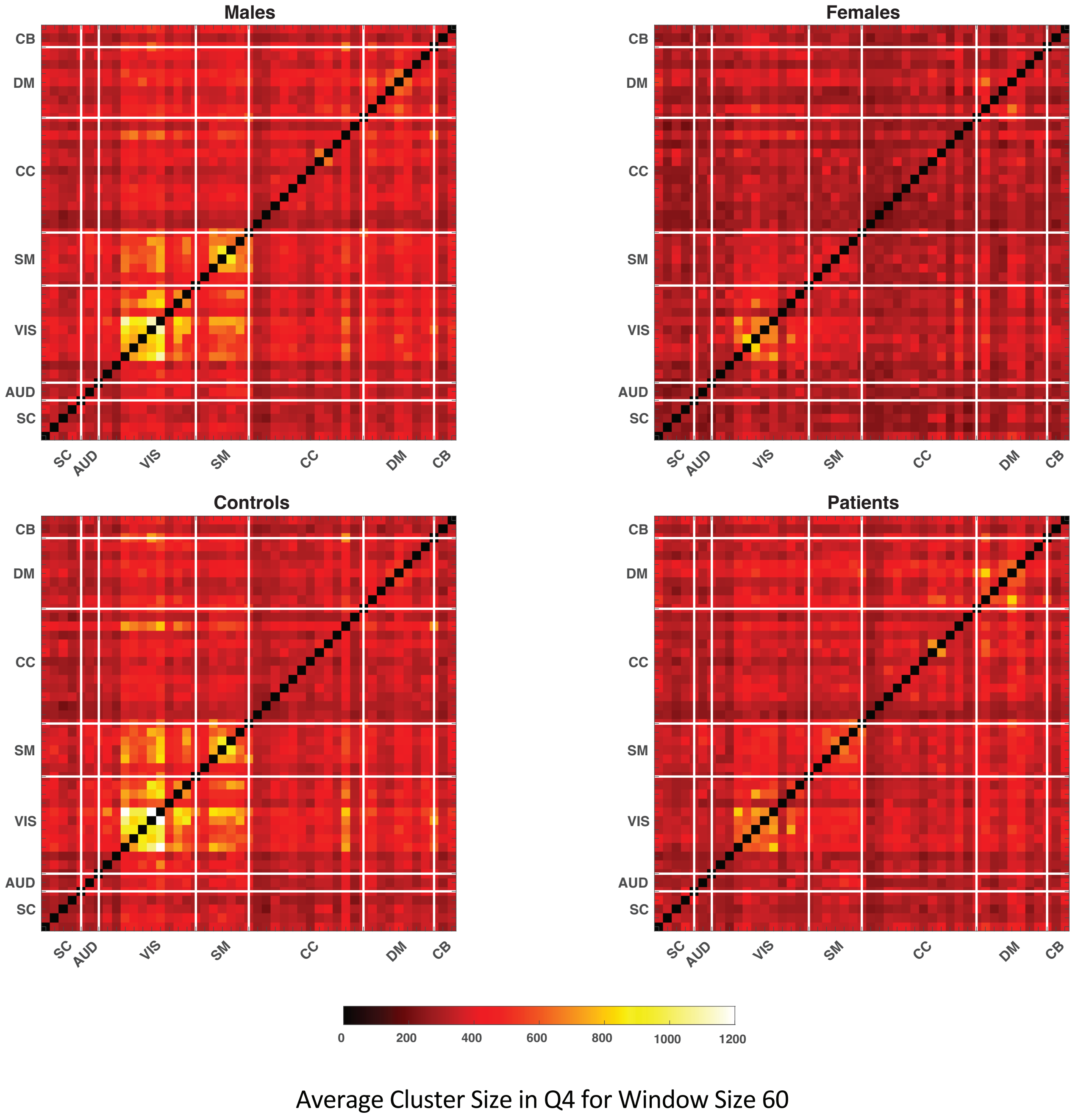

Figure S10. The average cluster size is calculated throughout all WW correlation maps for each quartile within the subgroups. In the figure, each cell of the matrices represents the average cluster size of a particular component pair for the subgroups. The matrices represent the average cluster size of the male, female, healthy controls and people with schizophrenia in the last quartile from A to D with window size 60TR. The thick white lines split the map into regions for seven distinct networks defined in the methods section

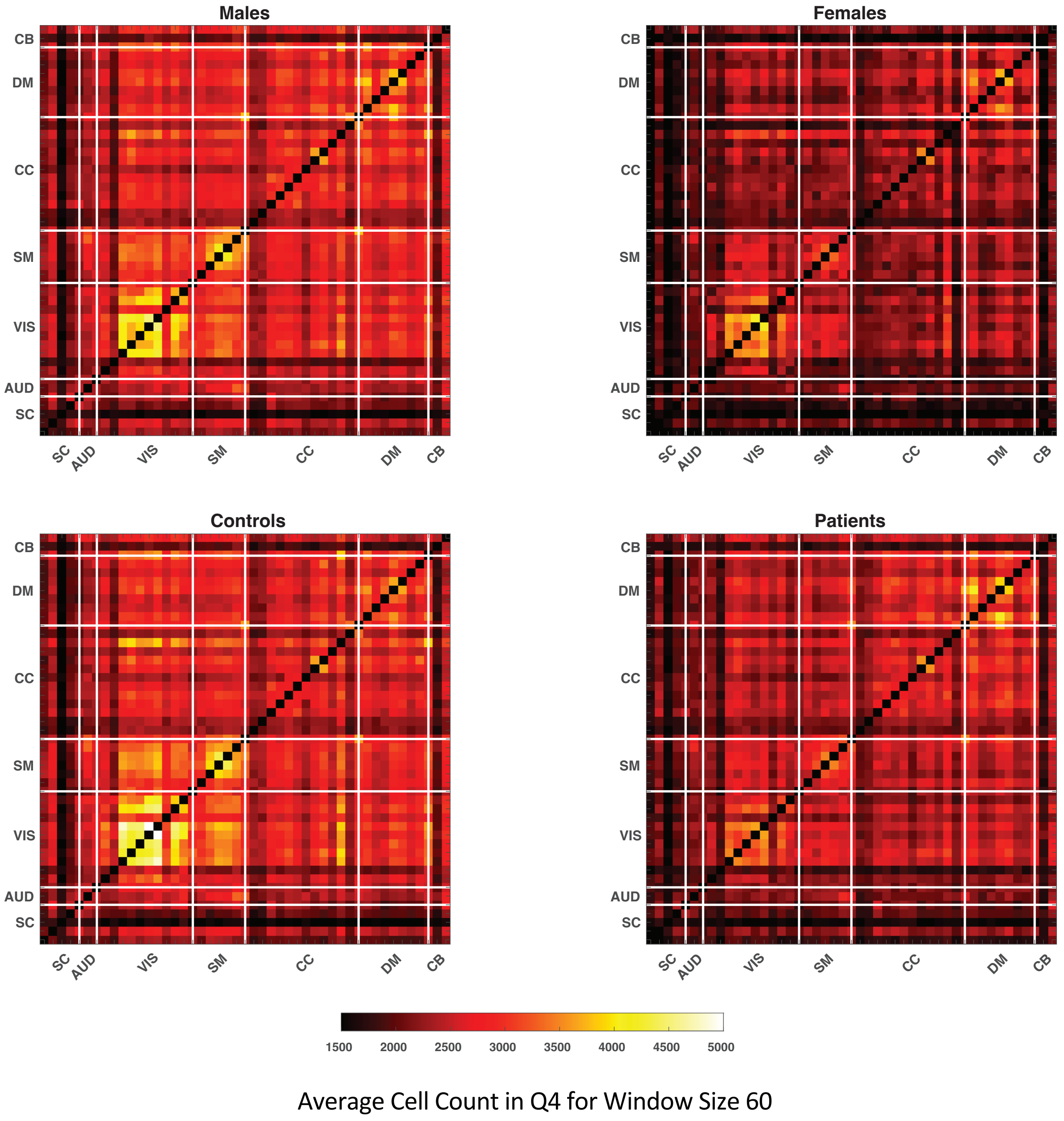

Figure S11. The average cluster size is calculated throughout all WW correlation maps for each quartile within the subgroups. In the figure, each cell of the matrices represents the average cell-count of a particular component pair for the subgroups. The matrices represent the average cluster size of the male, female, healthy controls and people with schizophrenia in the last quartile from A to D with window size 60TR. The thick white lines split the map into regions for seven distinct networks defined in the methods section
